# Effects of walnut oil on lipid profiles in hyperlipidemic type 2 diabetic patients: a randomized, double-blind, placebo-controlled trial

**DOI:** 10.1038/nutd.2017.8

**Published:** 2017-04-10

**Authors:** M J Zibaeenezhad, P Farhadi, A Attar, A Mosleh, F Amirmoezi, A Azimi

**Affiliations:** 1 Cardiovascular Research Center, Shiraz University of Medical Sciences, Shiraz, Iran; 2 Student Research Committee, Shiraz University of Medical Sciences, Shiraz, Iran; 3 Cardiovascular Research Center, TAHA Clinical Trial Group, Mohammad Rasool Allah Research Tower, Shiraz University of Medical Sciences, Shiraz, Iran

## Abstract

**Background:**

The role of herbal medicine is now well documented in preventing and controlling diabetes mellitus. The main aim of this study was to evaluate the effects of walnut oil consumption on lipid profiles of hyperlipidemic patients with type 2 diabetes.

**Methods:**

In a randomized, double-blind, placebo-controlled clinical trial, 100 hyperlipidemic type 2 diabetic patients aged 35–75 years were assigned to receive 15 cc Persian walnut oil or placebo every day for 90 days. The primary outcomes were the lipid profiles.

**Results:**

Consumption of walnut oil by type 2 hyperlipidemic diabetic patients resulted in a significant decrease in total cholesterol levels (treatment difference (TD)=−30.04, *P*<0.001), triglyceride (TG) level (TD=−15.04, *P*=0.021), low-density lipoprotein (LDL) level (TD=−30.44, *P*<0.001) and total cholesterol to high-density lipoprotein (HDL) ratio (TD=−0.72, *P*<0.001) compared to the control group. There was a trend toward increasing HDL level with consumption of walnut oil (TD=2.28, *P*=0.06). Frequency of patients reaching a LDL level below 100 was higher in the case group (20 vs 0%).

**Conclusions:**

Addition of walnut oil in the daily diet of type 2 diabetic patients improves lipid profiles. Thus, it may be associated with a coronary artery disease risk factor modulation. Also, walnut oil may serve as a helpful natural remedy for hyperlipidemic patients with type 2 diabetes.

## Introduction

Diabetes mellitus is characterized by an array of dysfunctions ensuing as consequences of hyperglycemia. In 2011, the International Diabetes Federation estimated that 336 million people had diabetes and the number of diabetics is expected to reach 552 million in 2030, which means one in ten adult will have diabetes by that time.^1^

It is very important as diabetics are at increased risk of many disease-related complications such as macrovascular complications. Coronary artery disease (CAD), a macrovascular disease, is the leading cause of death among diabetic patients.^2, [Bibr bib4]^ Indeed, CAD is one of many complications of atherosclerosis, which accounts for ~80% of mortality and 75% of hospitalizations associated with diabetes. Serum lipid abnormalities, which are often present in diabetics, accelerate initiation and development of atherosclerosis and dyslipidemia. In comparison with those without diabetes, diabetics have a higher serum triglyceride level and a lower high-density lipoprotein (HDL) cholesterol level.^5^ Hypertriglyceridemia facilitates generation of smaller and denser low-density lipoprotein (LDL) cholesterol particles, which enter the arterial wall more easily and could accelerate formation of atherosclerosis.^6^ Although the serum level of LDL does not differ significantly between diabetic and non-diabetic individuals, diabetic patients typically have smaller, denser and more atherogenic LDL particles.^2^ Increased preponderance of atherogenic LDL particles has become the primary target of lipid-lowering therapies in diabetic patients,^2, 7^ and therapeutic strategies focusing on lowering LDL levels (mainly by using statins) have significantly reduced the risk of CAD in diabetic patients.^8, 9^

Currently, diabetes is not curable and medications can only delay the progression of disease complications and reduce the blood glucose level, but it can be preventable. Therefore, primary prevention through modifications in diet and lifestyle is of high importance. Currently, it is suggested that including nuts in dietary regimen could play a significant role in prevention and management of diabetes complications.^10^

It is now well documented that nuts can improve blood-lipid profile and reduce the risk of CAD.^11, 12, 13^ Nuts have many nutritional benefits; besides having a favorable fatty acid profile, nuts are a rich source of bioactive compounds such as l-arginine, the precursor amino acid of the endogenous vasodilator nitric oxide,^14^ dietary fibers, phytochemicals, folic acid and antioxidants^15^ and it has been shown that frequent nut consumption or intake of more than 4–5 servings per week (one serving is 1 oz. or 12–14 halves or 1/4 cup of walnut) significantly reduces adjusted relative risk of CAD.^16, 17, 18, 19^

In comparison with other kinds of nuts, most nuts are high in monounsaturated fatty acids, whereas walnut is composed largely of polyunsaturated fatty acids, and it is the richest one in alpha-linolenic acid content,^20^ which gives it additional anti-atherogenic properties.^21^ Such a unique property distinguishes walnut from most other nuts, making it an interesting and intriguing target for investigation.

As the administration of the walnut oil instead of the whole nut is an easier way for the participants to take the ingredients in the study and also an easier way for standardization of walnut content for comparison between the study groups, we designed a study aiming at investigating the effects of walnut oil on lipid profiles of hyperlipidemic type 2 diabetic patients (T2DM).

## Materials and methods

### Trial design

This double-blind, placebo-controlled, randomized trial was performed on 100 hyperlipidemic patients with T2DM referring to the Shiraz Healthy Heart House during a 6-month period from November 2012 to April 2013. The Medical Ethics Committee of Shiraz University of Medical Sciences approved the protocol of the study, and a signed informed consent was obtained from each participant. The trial was registered at Iranian registry of clinical trials (www.IRCT.IR) under the registration number of IRCT2014030611375N2.

### Participants

All the participants were hyperlipidemic patients with T2DM recruited from Shiraz Healthy Heart Home in Shiraz, Iran. Eligible participants had a clinical diagnosis of T2DM for at least 2 years, were non-smokers, were aged 35–75 years, had serum plasma triglyceride level between 150 and 350 mg dl^−1^, had total cholesterol level more than 200 mg dl^−1^, LDL <160 or HDL <50 in females and <40 in males and were receiving maximum two oral antidiabetic medications. Exclusion criteria included a history of chronic or metabolic diseases, pregnancy, regular use of medication (fibrate and HMG-COA reductase inhibitor) or supplements known to affect the blood lipids, and currently receiving insulin therapy.

### Intervention

T2DM subjects were randomized into two study groups. Individuals in one group administered 4 walnut oil capsules containing 1.25 cc Persian walnut (Juglansregia L.) oil, three times daily with food (15 cc daily) for 90 days (Walnut oil group). In the other group, the individuals administered four placebo capsules containing 1.25 cc distilled water, three times daily with food (15 cc daily) for 90 days (placebo group). The patients in both groups were advised not to take any other forms of walnut/nut or change their diet style and habits. Persian walnut was cold pressed to extract the oil from them. Approximately 4 pounds (1.8 kg) of walnut was needed to make 1 liter of the oil. Both capsules were made by Shiraz Pharmacology School. We chose 15 ml of oil as its consumption corresponds to high amounts of walnut consumption (4–5 servings in a week). The shape and packing of walnut oil and placebo capsules were similar, so the patients and researchers were unable to detect which one was walnut oil or not. There were no measures to assess the adherence of patients to the intervention except self-declaration.

### Outcomes

The primary outcomes were lipid profiles changes including total cholesterol, triglyceride, HDL and LDL at the beginning and 3 months after the intervention. Collection and analyses of all clinical and laboratory data were performed by the study personnel blinded to group assignment.

### Measurements

The serum triglyceride was measured by glycerol phosphate oxidase-phenol and aminophenazone method providing a normal upper limit of 200 mg dl^−1^ (2.3 mmol^−1^). The total cholesterol was also checked by cholesterol oxidase/phenol and aminophenazone technique, which provided an upper limit normal value or 220 mg dl^−1^ (5.6 mmol^−1^). The HDL cholesterol was measured by dextran magnesium sulfate. The LDL cholesterol was derived according to the following formula: LDL=total cholesterol−(HDL+TG/5). Body mass index (BMI) was calculated using the weight and height measurements. Blood pressure was measured after a 5-min resting period using a standard mercury sphygmomanometer.

### Randomization

Randomization was done using a computer-based random digit generator based on the registration number of the patients (on the order of referral). Study capsules were allocated in separate packs blinded and labeled using a four-digit code. The information regarding which codes correspond to which treatment was maintained by the project coordinator. Apart from the project coordinator, the patients, the staff involved in clinical center, and members collecting and analyzing data were blinded to the intervention allocation.

### Statistical analysis

In order to have 90% power to detect the significant differences between the changes of lipid profiles, 40 patients were required in each study group (*P*<0.05, two-sided). The total sample size of 100 was selected for possible withdrawal of patients from the study. All statistical analyses were performed using the statistical Package for Social Sciences version 17.0 (SPSS Inc., Chicago, IL, USA). Baseline characteristics were analyzed using independent *t*-test or *χ*^2^ tests. The 95% confidence intervals for the means of data were calculated, and the significance of the differences between the results within groups was assessed using paired *t*-tests. A two-sided *P*-value <0.05 was considered statistically significant.

## Results

**Figure 1:**
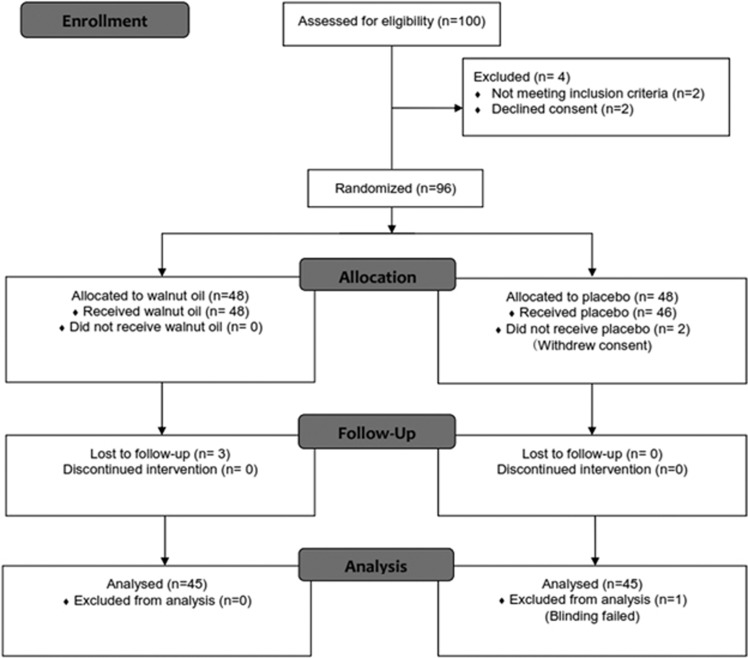
CONSORT 2010 flow diagram for this randomized, double-blinded, placebo-controlled clinical trial of effects of walnut oil on lipid profiles in hyperlipidemic type 2 diabetic patients.

Of the 100 patients who were assessed for eligibility, 96 underwent randomization. Two patients in the placebo group did not receive the assigned study intervention and three patients were lost to follow-up in the walnut oil group. Blinding failed for one of the patients of the placebo group, and finally 45 patients were enrolled in the walnut oil group and 45 individuals were enrolled in the placebo group (Figure 1).

The baseline characteristics of both groups are shown in Table 1. No significant differences were observed in baseline characteristics such as age, gender, BMI, blood pressure, fasting plasma glucose (FPG), hemoglobin A1c (HbA1c) and lipid profiles between the walnut oil and placebo groups.

The walnut oil was analyzed by Shiraz Pharmacology School and its analysis showed that walnut oil used for the study was rich in unsaturated fatty acids. It contained linoleic (64%), oleic acid (13.3%), α-linolenic acid (8.5%) and palmitoleic acid (0.2%). On the other hand, it contained some saturated fatty acids including palmitic acid (13.6%) and stearic acid (1.3%).

### Serum lipid profile

Compared to the control group, consumption of walnut oil resulted in a significant decrease in total cholesterol levels (treatment difference (TD)=−30.04, *P*<0.001), TG level (TD=−15.04, *P*=0.021), LDL level (TD=−30.44, *P*<0.001) and total cholesterol to HDL ratio (TD=−0.72, *P*<0.001). There was a trend toward increasing HDL level with consumption of walnut oil (TD=2.28, *P*=0.06; Table 2).

From 45 patients in the case group and 44 patients in the control group with a LDL level above 100, 9 patients in the case group (20%) and none in the control group (0%) reached a LDL level below 100 after 3 months.

### Side effects

There were two cases of mild gastrointestinal oil intolerance, none of whom discontinued the intervention. No other side effects were noticed.

## Discussion

These results of our clinical trial showed that addition of 15 ml walnut oil to previous diet of diabetics, without any change in the participants' previous diet for 90 days significantly decreased the total cholesterol, LDL cholesterol, triglyceride and total cholesterol to HDL ratio.

In our trial, the serum level of total cholesterol and LDL cholesterol was significantly decreased after 90 days of walnut oil consumption. Although cholesterol-lowering effects of walnut consumption is a well-established and well-documented finding,^11, 22, 23^ it is postulated that only high amounts of walnut consumption could exert such cholesterol-lowering effect, while lower and more practical levels of consumption did not affect the serum cholesterol, neither LDL nor total cholesterol.^24^ As shown, most of lipid-lowering and cardio-protective effects of walnut consumption are related to walnut oil.^25^ Walnut oil is composed largely of polyunsaturated fatty acids and incorporation of polyunsaturated fatty acids in the LDL composition facilitates receptor-mediated LDL clearance by hepatocytes, which could explain cholesterol-lowering effects of walnut consumption.^26^

According to the results of our study, serum TG levels were significantly decreased after the trial. This is consistent with two previous studies^23, 27, 28^ and in contrast with some others.^11, 22, 24, 29^ A study by Sabate *et al.*^30^ has revealed that 4-weeks of walnut consumption decreases TG, but not to a significant degree. Such diversity of observations may be due to one important limitation of our study as we had not controlled diet and energy intake of the participants during the trial. Consumption of walnut oil could have decreased the appetite of consumers in the walnut oil group, which not only affects their diet/energy intake but also disturbs all other measured and diet-dependent parameters. Duration of the trial could also explain the diversity, as in one parallel randomized controlled trial with three groups (receiving low fat, modified low fat and modified low fat inclusive of 30 g of walnuts per day) performed by Tapsell *et al.*,^29^ the serum TG levels were decreased at the first 3 months in all of the groups and no significant correlation was observed between walnut consumption and serum TG levels.

In our study, there was no significant difference in the serum HDL levels between the walnut oil and placebo groups. A meta-analysis consisting of 13 trials did not find any meaningful correlation between walnut consumption and serum HDL levels. In that meta-analysis, dietary interventions lasted from 4 to 24 weeks with an average of 6 weeks.^22^ On the other hand, a long-term survey (12-months) of effects of increased dietary walnut consumption revealed that 12-month consumption of walnuts (30 g per day) in the patients with type 2 diabetes could significantly increase the serum HDL levels in a time-dependent manner.^31^ This is not inconsistent with our findings as our trial was performed for a 3-month period. However, in that period, the total cholesterol level to HDL cholesterol ratio was significantly decreased which is consistent with previous study.^29^ It is shown that reduction in total cholesterol to HDL ratio is followed by an improvement in endothelial function.^32^ Therefore, it can be hypothesized that walnut oil consumption may contribute to improving endothelial function by reducing total cholesterol to HDL ratio.^33^

Previous studies have shown a strong correlation between insulin resistance and initiation of atherosclerosis.^5,3^ So it is important to know that walnut consumption not only has no negative impact on glycaemia inidices but also improves it. Previously, we had shown that the mean level of HbA1c was decreased significantly with walnut oil consuming.^34^ A finding which is consistent with those of Tapsell *et al.* as they have shown that long-term walnut consumption could significantly reduce the fasting plasma glucose and insulin levels.^31^ Other previous studies have not found any meaningful correlation between walnut consumption and level of HbA1c, FPG or glucose tolerance.^32, 33^

In our trial we noticed that most of our patients in both study arms had a high BMI (~27). As some studies documented, high BMI is strongly associated with type 2 diabetes and the prevalence of hyperlipidemia and hypertension increases with higher BMI.^35, 36, 37^ Our treatment had no effect on the patients' BMI. This is in agreement with previous data. In a meta-analysis by Banel and Hu,^22^ no significant weight change was noticed during short-term dietary interventions with walnuts.

There were a number limiting factors diminishing the impact of the results. First, we have not evaluated previous diets of the participants and we did not control their diet during the trial, which could have affected our findings, especially those about TG and HbA1c. One other limitation is that we did not evaluate the endothelial function, as it is affected by changes of TC/HDL ratio.^32^ In addition, another study showed that walnut consumption improves endothelium-dependent vasodilation in type 2 diabetic individuals.^33^ Finally, we did not evaluate the possible effects of walnut oil consumption on arterial stiffness, inflammation and platelet activation in diabetic patients.

In conclusion, we showed that addition of 15 ml walnut oil to previous diet of diabetics, for 90 days, significantly decreased the total cholesterol, LDL cholesterol, triglyceride and total cholesterol to HDL ratio, which are all important in management of diabetes. It can be hypothesized that these effects may have an impact on reduction of CAD risk of and other disease-related complications in diabetic patients. This assumption needs future studies to be confirmed. Finally, it can be concluded that walnut oil may serve as a helpful natural remedy in hyperlipidemic patients with type 2 diabetes.

## Figures and Tables

**Table 1:** Baseline characteristics of participants

*Characteristics*	*Walnut oil group (45)*	*Placebo group (45)*	P*-value*
Age (years)	55.5±10.7	54±11.4	0.51
Sex	–	–	0.09
Male (%)	25 (55.6)	28 (62.2)	–
Female (%)	20 (44.4)	17 (37.8)	–
BMI (Kg m^−2^)	27.6±2.4	27.2±2.3	0.43
Systolic blood pressure (mm Hg)	137.7±14.5	132.5±17	0.75
Diastolic blood pressure (mm Hg)	81.6±7.6	81.1±7.1	0.29
Mean blood pressure (mm Hg)	109.6±10.1	106.8±10.4	0.19
FPG (mg dl^−1^)	158.3±48.1	153.8±54.7	0.12
HbA1c (%)	7±1.1	6.9±1.2	0.89
			
*Lipid profiles*			
Total cholesterol (mg dl^−1^)	234.1±33	242±38.4	0.09
TG (mg dl^−1^)	194.5±28.2	208.1±45.7	0.55
LDL (mg dl^−1^)	144.1±22	147.8±35.1	0.34
HDL (mg dl^−1^)	48.3±10.2	46.1±11.8	0.68
Total cholesterol to HDL ratio	5±1.1	5.5±1.7	0.07
			
*Oral hypoglycemic agents*			
Metformin (%)	97.7	95.5	0.87
Sulfonylureas (%)	64.4	71.1	0.36
Glitazones (%)	6.6	4.4	0.53

Abbreviations: FPG, fasting plasma glucose; HbA1c, hemoglobin A1c; HDL, high-density lipoprotein; LDL, low-density lipoprotein; TG, triglyceride.

**Table 2:** Comparison of the changes in outcome measures before and after the intervention

*Characteristics*	*Walnut oil group (*n*=45)*			*Placebo group (*n*=45)*			*Treatment effect (%)*	*TD*	*95% confidence interval*	P*-value*
	*Baseline*	*3 months*	P*-value*	*Baseline*	*3 months*	P*-value*				
*Lipid profiles*										
Total cholesterol (mg dl^−1^)	234.1±33	207.7±39.7	<0.001	242.1±38.4	245.8±42.7	0.34	−13.40	−30.04	−42.82 to −17.26	<0.001
TG (mg dl^−1^)	194.5±28.2	173.1±31.4	<0.001	208.1±45.7	201.8±47.9	0.19	−8.13	−15.04	−27.78 to −2.30	0.021
LDL (mg dl^−1^)	144.1±22	117.4±31.8	<0.001	147.8±35.1	151.5±36.5	0.18	−21.62	−30.44	−39.14 to −21.73	<0.001
HDL (mg dl^−1^)	48.3±10.2	50.3±9.8	0.06	46.1±11.8	45.8±10.3	0.73	3.91	2.28	−0.14 to 4.72	0.06
Total cholesterol to HDL ratio	5±1.1	4.3±1.4	<0.001	5.5±1.7	5.6±1.5	0.9	−17.38	−0.72	−1.11 to −0.34	<0.001
